# Acute myeloid leukemia in a father and son with a germline mutation of *ASXL1*

**DOI:** 10.1186/s40364-018-0121-3

**Published:** 2018-02-13

**Authors:** Karen Seiter, Kyaw Htun, Paul Baskind, Zach Liu

**Affiliations:** 1 Department of Medicine, New York Medical College, Valhalla, USA; 2 Emerge Laboratories, Suffern, USA

**Keywords:** Familial acute myeloid leukemia, Familial myelodysplastic syndrome, *ASXL1*, Nextgen sequencing

## Abstract

**Background:**

Myelodysplastic syndromes and acute myeloid leukemia usually occur sporadically in older adults. More recently cases of familial acute myeloid leukemia and/or myelodysplastic syndrome have been reported.

**Case presentation:**

Currently we report a father and son who both developed myelodysplastic syndrome that progressed to acute myeloid leukemia. Both patients were found to have the identical mutation of *ASXL1* on nextgen sequencing of both hematologic and nonhematologic tissues.

**Conclusions:**

These cases support the diagnosis of a germline mutation of *ASXL1*.

## Background

Myelodysplastic syndromes and acute myeloid leukemia usually occur sporadically in older adults. These diseases also occur in younger patients with congenital syndromes such as Fanconi anemia, dyskeratosis congenita, and severe congenital neutropenia [1, 2]. More recently cases of familial acute myeloid leukemia (AML) and/or myelodysplastic syndrome (MDS) have been reported with mutations in *RUNX1*, *CEBPA*, *GATA2*, *ANKRD26*, *SRP72*, *DDX41* or ETV6 [1, 2]. Currently we report a father and son who both developed MDS to AML. Both patients were found to have the identical mutation of *ASXL1* on nextgen sequencing. The presence of the mutation in nonhematologic tissues supports the diagnosis of a germline mutation of *ASXL1*.

## Case presentation

### Son

A 46 year old man with no prior medical history was referred to us for evaluation of leucopenia and thrombocytopenia in June of 2012. The white blood count was 3200/mm^3^ with 51% neutrophils, 46% lymphocytes, 2% monocytes and 1% eosinophils. The hemoglobin was 13.1 g/dl and the platelet count was 103,000/mm^3^. The bone marrow was normocellular with 20% blasts and megakaryocytic dysplasia. Flow cytometry demonstrated that the immunophenotype was CD13, CD33, CD11c, CD34, CD117, HLA-DR, CD71, CD41 (very dim), CD38, and CD9 (dim). Cytogenetics were normal, however only 4 metaphases were obtained. AML and MDS fluorescence in-situ hybridization (FISH) panels were normal. A diagnosis of high grade MDS evolving to AML was made.

The patient received induction therapy with high dose cytarabine, high dose mitoxantrone, and etoposide. Bone marrow evaluation upon count recovery showed a hypercellular marrow with 5% myeloblasts and dysplastic features, including pseudo-Pelger-Huet cells and dyserthropoiesis. The patient received a second cycle of chemotherapy with high dose cytarabine. The bone marrow then showed a normocellular marrow with maturing trilineage hematopoiesis, dyserthropoiesis and 1% blasts.

In September 2012 the patient underwent an allogeneic stem cell transplant using a fludarabine, melphalan, busulfan conditioning regimen and peripheral blood stem cells from his HLA identical sister. He has remained well, with full donor engraftment, for the past 3.5 years.

### Father

In September 2016 the patient’s 75 year old father presented to us. He had been diagnosed with MDS elsewhere in January 2012. The initial bone marrow demonstrated 5% blasts and normal cytogenetics. He had received decitabine for 4.5 years. The patient was referred to us after his blood counts had worsened. The white blood count was 2900/mm^3^ with 56% neutrophils, 42% lymphocytes, 1% monocytes, and 1% eosinophils. The hemoglobin was 9.6 g/dl and the platelet count was 28,000/mm^3^. Bone marrow evaluation demonstrated AML with MDS-related changes, with 26% blasts and an immunophenotype of CD13, CD33, CD34, CD117, and HLA-DR. Cytogenetics were complex with 44,XY,-3,del(5)(q13q31),add(9)(q13),-10,der(16)t(9;16)(q13;q11.2),-17,+ 21[7]/46,XY[6]. Mutations in FLT3 and NPM were not detected on PCR. The patient received CLAG-idarubicin chemotherapy but had refractory disease. He then opted for hospice care and expired shortly thereafter.

Nextgen sequencing was performed on the diagnostic bone marrow sample and a buccal swab from the father, and on peripheral blood, buccal swab, skin biopsy and archived bone marrow from the son. Genomic DNA was extracted and purified. Targeted sequencing was performed by using a combination of multiplexed PCR (AmpliSeq Hotspot primers) to generate libraries. Adapters were then ligated to the PCR products, where the sequences were tagged with specific barcodes. The barcoded libraries were then clonally amplified using emulsion PCR (emPCR). The emPCR was then purified using magnetic bead purification followed by semiconductor-based sequencing on an Ion Torrent PGM (Life Technology). The targeted gene panel developed in this laboratory includes the following 32 genes: *ABL1, ASXL1, BRAF, CBL, CDKN2A, CEBPA, CREBBP, CSF1R, DNMT3A, ETV6, EZH2, FBXW7, FLT3, HRAS, IDH1, IDH2, JAK3, KIT, KRAS, NORCH1, NPM1, NRAS, PDGFRA, PHF6, PTEN, RUNX1, SF3B1, SRSF2, TET2, TP53, U2AF1, and WT1*. Each variant was analyzed manually using variant caller from Ion Torrent software (Life Technology) and cross-referenced with Ingenuity(tm) software (Qiagen) for bioinformatics. Evidence based categorization of the variants were performed by genomic analysis software (GenomOncology). This test is designed to detect alterations in genes that are clinically known to play a role in tumor genesis and provide prognostic value. For each gene, the minimum required coverage is 500 sequence reads based on bidirectional sequencing. The minimum acceptable frequency is 5%.

Both patients were enrolled on a clinical trial of familial leukemias that was approved by the Committee on the Protection of Human Subjects at New York Medical College. Both specifically consented to genetic testing. These included consent for publication.

Results of nextgen sequencing are summarized in Table 1. The father’s diagnostic bone marrow demonstrated a mutation in *ASXL1* (c.2957A > G; p.N986S) with an allelic frequency of 50%. In addition a mutation in TP53 was detected with an allelic frequency of 12%. A buccal swab identified the same *ASXL1* mutation at a frequency of 49.4%. We then tested the patient’s son for the same panel of mutations. His peripheral blood did not exhibit any mutations, however the blood was derived from his healthy sister (100% XX by FISH at the time of peripheral blood nextgen sequencing). A buccal swab on the son demonstrated the identical *ASXL1* mutation (c.2957A > G; p.N986S) at a frequency of 21.7%. A skin biopsy demonstrated this mutation at a frequency of 41.6% and his archived first post chemotherapy bone marrow (with 5% blasts) demonstrated the mutation at a frequency of 50.5%.

**Table 1:** Nextgen sequencing results

Patient	Date	Source	Gene	Mutation	Allelic burden
Father	9/20/2016	Bone marrow	TP53 ASXL1	c.675delT; p.G226 fs c.2957A > G; p.N986S	12.0% 50.0%
	10/26/2016	Buccal swab	ASXL1	c.2957A > G; p.N986S	49.4%
Son	7/17/2012	Bone marrow (5% blasts)	ASXL1	c.2957A > G; p.N986S	50.5%
	11/4/2016	Buccal swab	ASXL1	c.2957A > G; p.N986S	21.7%
	12/09/2016	Peripheral blood (100% donor)	Normal		
	1/13/2017	Skin biopsy	ASXL1	c.2957A > G; p.N986S	41.6%

## Discussion and conclusions

Constitutive mutations of *ASXL1* occur in the Bohring-Opitz syndrome, a rare condition characterized by facial anomalies, multiple malformations, severe intellectual disabilities and early death [3]. Somatic mutations of *ASXL1* were first reported in patients with hematologic malignancies in 2009 [4]. Subsequent studies have since shown that mutations in *ASXL1* occur in approximately 6% to 30% of patients with AML and in 15 to 20% of patients with MDS [5–7]. These mutations are more common in older patients and are more common in patients with secondary rather than de novo AML [8]. Mutations in *ASXL1* have an adverse effect on survival in both MDS and AML [5, 9, 10].

In a mouse model developed by Abdel-Wahab, mice with germline complete deletion of *ASXL1* (*ASXL1*^*−/−*^) were no longer viable by day 19.5 and exhibited microphthalmia/anophthalmia, cleft palates and multiple skeletal abnormalities [11]. Mice with hematopoietic –specific deletion of *ASXL1* developed progressive leucopenia and anemia that was accompanied by an increase in erythroid precursor cells in both the bone marrow and spleen. Wang also reported that *ASXL1*^*−/−*^ mice had developmental abnormalities including dwarfisim, anophthalmia, and 80% embryonic lethality [12]. Surviving mice developed features of MDS. In this model, *ASXL1*^*+/−*^ mice also developed an MDS-like phenotype indicating a haploinsufficient effect of *ASXL1* in the pathogenesis of myeloid malignancies.

*ASXL1* mutations have been reported in a limited number of patients with familial hematologic malignancies. Somatic mutation of *ASXL1* was reported in a patient with chronic myelomonocytic leukemia in the setting of a germline mutation in *ANKRD26* [13]. Somatic mutations of *ASXL1* have also been reported in patients with germline mutations of *GATA2* [14]. Hamadou described two sisters with NHL who were noted to have a presumed germline *ASXL1* mutation in peripheral blood samples performed when they were in remission [15]. The sisters’ healthy mother and brother also carried this mutation.

Our two patients shared the identical *ASXL1* mutation in multiple tissues. This specific mutation (c.2957A > G; p.N986S) has been reported in one case of Bohring-Opitz syndrome in the National Center for Biotechnology Information database. Its significance in hematologic malignancies to date is unclear, however it is a missense mutation in exon 12, a site of other known pathogenetic mutations in *ASXL1* in hematologic malignancies^14^. Both patients were similar to the heterozygous mouse models in that their hematologic malignancies occurred after a prolonged latency period. The son’s earlier development of MDS/AML could be an example of anticipation, as is often seen in hereditary cancer syndromes. We believe this is the first reported case of a germline mutation of *ASXL1* in association with MDS and AML.

## Abbreviations

AML: Acute myeloid leukemia
CLAG: Cladribine, cytarabine, filgrastim
DNA: Deoxyribonucleic acid
FISH: Fluorescence in-situ hybridization (FISH)
HLA: Human leukocyte antigen
MDS: Myelodysplastic syndrome
PCR: Polymerase chain reaction
